# Drug-induced thrombocytopenia associated with trastuzumab in a patient with HER2-positive recurrent gastric cancer

**DOI:** 10.1007/s13691-021-00520-z

**Published:** 2021-11-02

**Authors:** Yuko Takano, Satoshi Furune, Yuki Miyai, Sachi Morita, Megumi Inoue, Tomoya Shimokata, Mihoko Sugishita, Ayako Mitsuma, Osamu Maeda, Yuichi Ando

**Affiliations:** Department of Clinical Oncology and Chemotherapy, Nagoya University Hospital, Nagoya, Japan

**Keywords:** Trastuzumab, Drug-induced thrombocytopenia, DITP, Gastric cancer

## Abstract

Here, we report a 57-year-old female patient with HER2-positive recurrent gastric cancer who experienced drug-induced thrombocytopenia associated with trastuzumab, a humanized anti-HER2 monoclonal antibody. Shortly after the initiation of S-1, oxaliplatin, and trastuzumab chemotherapy, the patient experienced severe thrombocytopenia and did not respond to platelet transfusions. Based on the findings of increased numbers of polynuclear megakaryocytes in the bone marrow and an elevated level of platelet-associated IgG (PA-IgG), the patient was diagnosed with drug-induced thrombocytopenia (DITP). The platelet count recovered rapidly with oral prednisolone (1 mg/kg). Since we initially suspected oxaliplatin as the causal agent, S-1 was restarted as a monotherapy, followed by trastuzumab after a 3-week interval, without oxaliplatin. On the second day after the addition of trastuzumab, severe thrombocytopenia occurred again, which suggests that trastuzumab was responsible for the DITP. The patient no longer experienced severe thrombocytopenia during the subsequent S-1 and oxaliplatin chemotherapy, which supports this hypothesis.

## Introduction

The humanized anti-human epidermal growth factor 2 (HER2) monoclonal antibody trastuzumab is a key drug in first-line chemotherapy regimens for HER2-positive advanced gastric or gastro-esophageal junction cancers. Trastuzumab occasionally causes hypersensitivity reactions, also known as infusion reactions, and latent or overt cardiac dysfunction after long-term treatment, whereas this drug rarely causes hematological toxicity or myelosuppression, which are common among cytotoxic anticancer drugs. Here, we report a case of acute, severe thrombocytopenia that was likely associated with trastuzumab.

## Case presentation

**Fig. 1:**
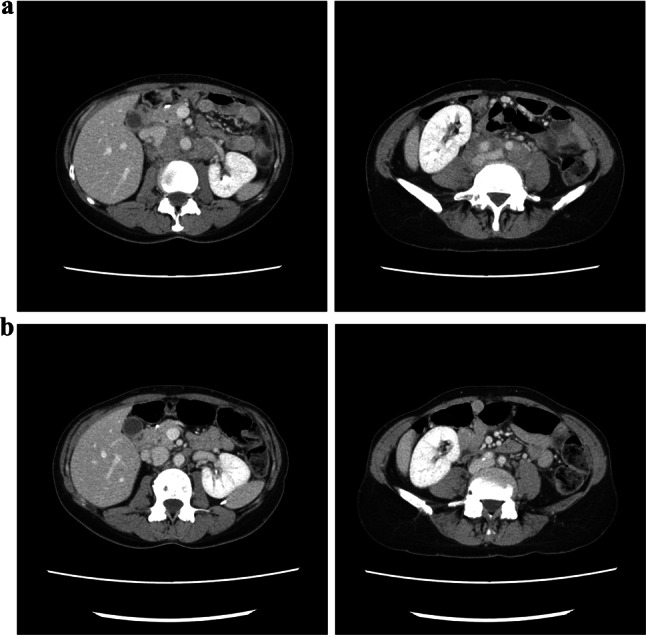
Abdominal CT image prior to treatment (**a**) and after the second infusion of trastuzumab (**b**). The para-aortic lymphadenopathy shrunk remarkably after treatment

A 57-year-old female patient was admitted to our hospital for swollen para-aortic lymph nodes detected by abdominal ultrasonography during a routine medical checkup. Thirteen years earlier, the patient had undergone total gastrectomy for T1bN1M0 Stage IB (AJCC 7th edition) gastric cancer. The patient did not receive adjuvant chemotherapy. She was also treated with entecavir for chronic hepatitis B and denosumab for osteoporosis and had received mecobalamin as an oral vitamin B12 supplement after total gastrectomy. *H. pylori* infection was not assessed. She also had no history of allergic reaction to medications or substances. A pathologic examination of CT-guided needle biopsy specimens from the para-aortic lymph nodes revealed HER2-positive moderately differentiated adenocarcinoma that was consistent with the metastatic lymph nodes from gastric cancer 13 years earlier (Fig. 1a). The patient was then diagnosed with HER2-positive recurrent gastric cancer.

The patient began treatment with first-line chemotherapy consisting of oral S-1 (a combination of tegafur, gimeracil, and oteracil potassium) at a dose of 50 mg twice daily on a 2-week on and 1-week off schedule, intravenous oxaliplatin (100 mg/m^2^), and trastuzumab (8 mg/kg as a loading dose followed by 6 mg/kg; CT-P6, Nippon Kayaku, Tokyo, Japan) given on day 1 of a 3-week cycle. Platelet count was 293,000/µL, and the other blood chemistry and hematologic findings were within the normal range at the time of chemotherapy initiation. Oxaliplatin and trastuzumab infusions were successfully completed; however, a grade 1 infusion reaction occurred during trastuzumab infusion. The patient was discharged the following day (day 2). At a follow-up outpatient visit on day 8, purpura and petechiae that appeared shortly after discharge were observed throughout the patient's body. She was also aware of macrohematuria that was persistent since day 3. Hematologic tests revealed grade 4 thrombocytopenia, a platelet count of 1000/µL, and moderate anemia with a hemoglobin level of 8.1 g/dL, while other blood chemistry and hematologic findings, such as white blood cell counts and neutrophil to lymphocyte ratio, were within normal range. According to the patient and her family, S-1 had been suspended since day 2.

**Fig. 2:**
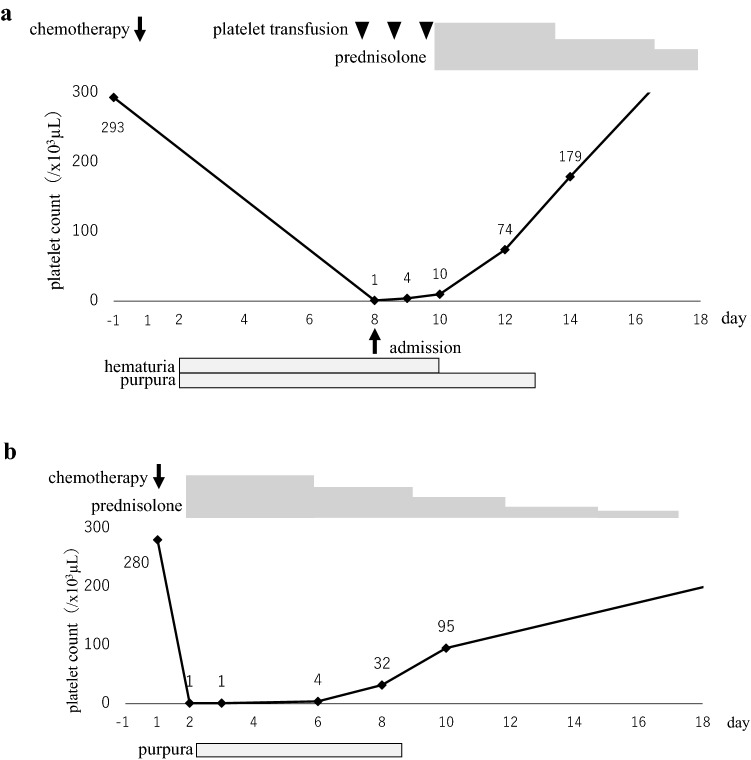
Changes in platelet count following the first (**a**) and second (**b**) trastuzumab infusions. Prednisolone was initiated at 1 mg/kg/day, slowly tapered, and discontinued

The patient was hospitalized immediately and received repeated platelet transfusions, but her platelet count did not recover (Fig. 2a). Based on the findings of increased numbers of polynuclear megakaryocytes in the bone marrow and an elevated level of platelet-associated IgG (PA-IgG, 96 ng/10^7^ cells; normal range is less than 46 ng/10^7^ cells), the patient was diagnosed with drug-induced thrombocytopenia (DITP). After starting oral prednisolone (1 mg/kg) on day 10, the platelet count recovered rapidly: 74,000/µL on day 12 and 179,000/µL on day 14. Since we initially suspected oxaliplatin as a causal drug, S-1 was restarted as a monotherapy, followed by trastuzumab at a dose of 8 mg/kg after a 3-week interval, without oxaliplatin. No infusion reaction was observed during or immediately after trastuzumab infusion, and the administration was completed safely. However, on the following day, purpura and petechiae reappeared throughout the patient's body, and hematologic tests revealed severe thrombocytopenia with a platelet count of 1000/µL, which suggests that trastuzumab was responsible for the DITP (Fig. 2b). Since a significant tumor response to treatment was recognized on follow-up CT imaging (Fig. 1b), the patient continued S-1 and oxaliplatin treatment and no longer experienced severe thrombocytopenia.

## Discussion

DITP is a rare adverse event associated with antibody-mediated platelet destruction that is triggered by exposure to a causal drug. [1–3] Among several proposed mechanisms of DITP, it is highly probable that autoantibodies specific for glycoproteins (IIb/IIIa) on the platelet surface caused the destruction of platelets in the circulation of this patient. Besides the elevated levels of PA-IgG, [4] trastuzumab re-challenge immediately reproduced thrombocytopenia, which is consistent with the immune mechanism of DITP.

More than 300 drugs and agents, including oxaliplatin [5, 6], have been reported to cause DITP [1–3]. Thirteen cases of trastuzumab-related DITP have been reported thus far, including the present case (Table 1) [7–19]. The present case differs from other reported cases in that our patient had gastric cancer, and to our knowledge, this is the only case treated with the trastuzumab biosimilar CT-P6. According to the manufacturer (Nippon Kayaku, Tokyo, Japan), no incidents of DITP associated with this trastuzumab biosimilar have been reported other than our case. It is unknown whether biosimilar exhibit cross-reactivity to reference trastuzumab.

**Table 1:** List of published cases of drug-induced thrombocytopenia caused by trastuzumab

Authors	Age	Sex	Cancer type	Number of cycles at onset	Concurrent chemotherapy	Platelet nadir (/×10^3^ μL)	Management
Cathomas et al. [7, 9]	54	F	MBC	1	None	3	IVIGs, steroids
Parikh et al. [8]	56	F	EBC	1	None	2	IVIGs
Jara Sánchez et al. [10]	37	F	MBC	1	Docetaxel, carboplatin	3	PLT, IVIGs, steroids, splenectomy
Drudi et al. [11]	Not reported	F	MBC	1	Docetaxel	7	PLT, IVIGs, steroids
Mantzourani et al. [12]	56	F	EBC	1	None	5	IVIGs
Aguirre et al. [13]	63	F	MBC	Not reported	Paclitaxel	22	Steroids
Pino et al. [14]	70	F	MBC	2	Vinorelbine	0	PLT, IVIGs, steroids
Zeng et al. [15]	57	F	EBC	21	Nab-paclitaxel, paclitaxel, carboplatin	28	Etamsylate, TPO
Miarons et al. [16]	70	F	EBC	4	Docetaxel	39	Steroids
Luo et al. [17]	39	F	EBC	2	Nab-paclitaxel	3	PLT, IL-11
Zhou et al. [18]	35	F	MBC	8	Capecitabine	1	TPO, PLT, steroids
Wang et al. [19]	52	F	EBC	1	None	1	PLT, steroids
Present case	57	F	Gastric	1	Oxaliplatin, S-1	1	PLT, steroids

*MBC* metastatic breast cancer, *EBC* early breast cancer, *PLT* platelet transfusion, *IVIG* intravenous immunoglobulin, *TPO* thrombopoietin

In conclusion, we report the first case of DITP associated with a trastuzumab biosimilar in a patient with gastric cancer, which provides useful insight into the practical management of DITP in molecular-targeted cancer treatment.
